# Editorial: Hierarchical Object Representations in the Visual Cortex and Computer Vision

**DOI:** 10.3389/fncom.2015.00142

**Published:** 2015-11-20

**Authors:** Antonio J. Rodríguez-Sánchez, Mazyar Fallah, Aleš Leonardis

**Affiliations:** ^1^Intelligent and Interactive Systems, Department of Computer Science, University of InnsbruckInnsbruck, Austria; ^2^Visual Perception and Attention Laboratory, Centre for Vision Research, School of Kinesiology and Health Science, York UniversityToronto, ON, Canada; ^3^School of Computer Science, University of BirminghamBirmingham, UK

**Keywords:** computer model, neurophysiology, computer vision, visual cortex, computational neurosciences

Over the past 40 years, Neurobiology and Computational Neuroscience have proved that deeper understanding of visual processes in humans and non-human primates can lead to important advancements in computational perception theories and systems. One of the main difficulties that arises when designing automatic vision systems is developing a mechanism that can recognize—or simply find—an object when faced with all the possible variations that may occur in a natural scene, and with the ease of the primate visual system. The area of the brain in primates that is dedicated to analyzing visual information is the visual cortex. The visual cortex performs a wide variety of complex tasks by means of seemingly simple operations. These operations are applied to several layers of neurons organized into a hierarchy, the layers representing increasingly complex, abstract intermediate processing stages.

In this research topic we propose to bring together current efforts in Neurophysiology and Computer Vision in order to better understand (1) How the visual cortex encodes an object from a starting point where neurons respond to lines, bars or edges to the representation of an object at the top of the hierarchy that is invariant to illumination, size, location, viewpoint, rotation and robust to occlusions and clutter; and (2) How the design of automatic vision systems benefits from that knowledge to get closer to human accuracy, efficiency and robustness to variations. In fact, the primate visual system has influenced computer vision systems for decades now since Hubel and Wiesel (1968) simple and complex cells inspired the Neocognitron (Fukushima, 1980). Since then, studies about the primate and human visual systems led the way to many more works on biologically-inspired computational vision, such as Tsotsos et al. (1995); Olshausen and Field (1996); Booth and Rolls (1998); Riesenhuber and Poggio (1999); Rodríguez-Sánchez and Tsotsos (2011), to name a few.

The answers to these issues bring hypotheses that are partially addressed in this research topic, raising additional new questions:

1. What are the mechanisms involved in these visual architectures? What are the limitations of feedforward connections? When is feedback and top-down priming necessary? The classical way of seeing feedback connections is for the enhancement of neural responses through top-down attentive processes (Moran and Desimone, 1985; Rodríguez-Sánchez et al., 2006; Perry et al., 2015). But lately, other studies support a role of feedback connections related to cell selectivity through recurrent networks (Neumann and Sepp, 1999; Angelucci and Bressloff, 2006).
2. The ventral stream areas (V1, V2, V4, inferotemporal cortex) have usually been considered to be the ones involved in object recogntion and the subject of several existing models (Serre et al., 2006; Rodríguez-Sánchez and Tsotsos, 2012). But, also recently, there are new findings that relate the dorsal stream with that same task (Konen and Kastner, 2008; Perry and Fallah, 2012). What are the differences between how objects are processed in the ventral and the dorsal streams? Which areas are involved in recognition and which in localization?
3. And finally, how much is learned and how much is genetically implemented (Rodríguez-Sánchez and Piater, 2014)? Even more, what is the relation between learning, sparse coding, selectivity and diversity (Olshausen and Field, 1996; Xiong et al., 2015) and how different learning strategies compare?

We present a total of 19 papers related to those questions. The following five papers deal with the questions related to visual architectures and their mechanisms. Ghodrati et al. (2014) studied whether recent relative successes in object recognition on various image datasets based on sparse representations applied in a feedforward fashion represented a breakthrough in invariant object recognition. In their study they showed, using a carefully designed parametrically controlled image database consisting of several object categories, that these approaches fail when the complexity of image variations is high and that their performance is still poor compared to humans. This suggests that learning sparse informative visual features may be one of the necessary components but definitely not a complete solution for a human-like object recognition system. A classical feedforward filtering approach is also challenged in the paper by Herzog and Clarke (2014), where the authors provided ample evidence, stemming from experiments from crowding research, to support their arguments that the computations are not purely local and feedforward, but rather global and iterative. On the same topic, Tal and Bar (2014) explored the role of top-down mechanisms which bias the processing of the incoming visual information and facilitate fast and robust recognition. This work specifically addresses the question of what happens to initial predictions that eventually get rejected in a competitive selection process. The work by Marfil et al. (2014) brings into focus another important aspect of biological visual sytems, namely attention. The authors studied a bidirectional relationship between segmentation and attention processes. They presented a bottom-up foveal attention model that demonstrates how the attention process influences the selection of the next position of the fovea and how segmentation, in turn, guides the extraction of units of attention. In Han and Vasconcelos (2014) the authors also researched the role of attention models, but this time in connection to object recognition. Using their recognition model, hierarchical discriminant saliency network (HDSN), they clearly demonstrated the benefits of integrating attention and recognition.

We provide an interesting discussion on the role of ventral and dorsal streams with a total of 10 articles. Kubilius et al. (2014) discusses the importance of surface representation and reviews recent work on mid-level visual areas in the ventral stream. We include here two models of shape related to those intermediate visual areas. The first approach is a recurrent network that achieves figure-ground segregation by assigning border ownership through the interaction between feedforward and feedback inputs (Tschechne and Neumann, 2014). The second approach is a trainable set of shape detectors that can be applied as a filter bank to recognize letters and keywords as well finding objects in complex scenes (Azzopardi and Petkov, 2014). The question that arises regarding computational models is of course, how faithful they are? This is what Ramakrishnan et al. (2015) answers by comparing the fMRI responses from 20 subjects to two different types of computer vision models: the classical bag of words and the biologically-inspired HMAX. HMAX is also the subject of study in Zeman et al. (2014), here the authors use that model to compare the robustness of complex cells to simple cells in the Müller-Lyer illusion. The final stage in the object recognition pathway is the inferotemporal cortex (IT), Leeds et al. (2014) present an fMRI study that tries to answers the problem of how starting from simple edge-like features in V1 we obtain neurons at the top of the hierarchy that respond to complex features as parts, textures or shapes. Using feed-forward object detection and classification modeling, Khosla et al. (2014) developed a neuromorphic system that also efficiently produces automated video object recognition. However, the visual system is not limited to only detecting objects, but can also detect the spatial relationships between objects and even between parts of the same object. The dorsal stream areas are thus also important for object representation with a focus on action via effectors such as the eyes or the hand. Theys et al. (2014) reviews how 3D shape for grasping is processed along the dorsal stream, focusing on the representations in the anterior intraparietal area (AIP) and ventral premotor cortex (PMv). Rezai et al. (2014) advances this by modeling the curvature and gradient input from the caudal intraparietal area (CIP) to visual neurons in AIP, using superquadric fits—used in robotics for grasp planning—or Isomap dimension reductions of object surface distances. They found that both models fit responses from primate AIP neurons. However, Isomaps better approximated the feedforward input from CIP making it the more promising model of how the dorsal stream produces shape representations for grasping. Yet the features used for grasping are only a subset of an object's features. While the integration of features along the ventral stream to form object representations is well-known, Perry and Fallah (2014) review recent findings supporting dorsal stream object representations and propose a framework for the integration of features along the dorsal stream.

Finally, four papers address the problem of learning and sparse coding. Rinkus (2014) shows that a hierarchical sparse distributed code network provides the foundation for the storage and retrieval of associative memory on top of building up an object representation. The end point of object processing is recognition, which the human visual system is very efficient at and many computational models are based upon. Webb and Rolls (2014) investigated how recognition of the identity of individuals and their poses can be separated. They showed that a model of the ventral visual system using temporal continuity, VisNet, can through learning develop pose-specific and identity-specific representations that are invariant to the other factor. In their biologically inspired study, Kermani Kolankeh et al. (2015) researched different computational principles (sparse coding, biased competition, Hebbian learning) capable of developing receptive fields comparable to those of V1 simple-cells and discovered that methods which employ competitive mechanisms achieve higher levels of robustness against loss of information which may be important to achieve better performance on classification tasks. While these studies have focused on using biologically-inspired visual processing in computational models, Bertalmío (2014) worked in reverse by taking an image processing technique used for local histogram equalization and applying it to a neural activity model. The resultant model predicts spectrum whitening, contrast enhancement and lightness induction, all behavioral aspects of visual processing. Time will tell if neuronal studies bear out this process.

We are bringing together two seemingly different disciplines: Neuroscience and Computer Vision. We show in this research topic that each one can benefit from the other. The latter can aid Neuroscience for testing hypotheses regarding the visual cortex in a non-invasive way, or otherwise when we reach technical limitations, e.g., how the information flows along the visual architectures (see Rodríguez-Sánchez, 2010 for a recent example). On the other hand, Computer Vision can benefit from Neuroscience in order to develop better, more robust, efficient and general systems than the ones present to date (Krüger et al., 2013).

Due to the complexity of vision (Tsotsos, 1987), objects/locations are considered to *compete* for the visual system's resources. The studies presented here show that—among other aspects—feedforward hierarchies are insufficient, supporting the need for top-down priming or attention. The interaction between feedforward and feedback inputs have an impact in neural encoding as shown in the models presented in this research topic. Not only competition, sparsity is another important mechanism. The aim is achieving efficient codes that represent and store object classes efficiently into memory since not every possible combination of features/parameters is feasible to be stored. Finally, a number of studies stress on the importance of the dorsal stream in shape and identity-object representation in order to interact with specific objects, e.g., grasping.

## Conflict of interest statement

The authors declare that the research was conducted in the absence of any commercial or financial relationships that could be construed as a potential conflict of interest.
